# The design of an estimation norm to assess nurses required for educational and non-educational hospitals using workload indicators of staffing need in Iran

**DOI:** 10.1186/s12960-018-0309-5

**Published:** 2018-08-23

**Authors:** Ali Vafaee-Najar, Mohammadreza Amiresmaeili, Mahmoud Nekoei-Moghadam, Seyed Saeed Tabatabaee

**Affiliations:** 1 Social Determinants of Health Research Center, Mashhad University of Medical Sciences, Mashhad, Iran; 2 Department of Health Management, Policy and Economic, Kerman University of Medical Sciences, Kerman, Iran; 3 Department of Health Management, Policy and Economic, Research Center for Health Services Management, Kerman University of Medical Sciences, Kerman, Iran

**Keywords:** Nurse, Workload, Hospital, Iran

## Abstract

**Background:**

One of the effective strategies in the fair distribution of human resources is the use of estimation norm of human workforce. A norm is a coefficient or an indicator for estimating the required human resources in an organization. Due to the changes in the available working hours of nurses in recent years and to use of a standard method, the Iranian Ministry of Health decided to update nursing estimation norm in hospitals in 2014–2015. This study aimed to design a nurse-required estimation norm for educational and non-educational hospitals based on the workload indicator in Iran.

**Methods:**

This was a descriptive cross-sectional study, carried out from December 2015 to November 2016 in 49 wards in 12 educational and 17 non-educational hospitals in Mashhad, Iran. The wards and hospitals who had the best performance in nursing care quality indicators were selected. Focus group, work study, consensus, interview, and reviewing documents, staff and patient records, and the calculations of modified Workload Indicators of Staffing Needs (WISN) were used to collect the data.

**Results:**

Patient care, cardiopulmonary resuscitation, and transfer out of the hospital were identified as the main activities of holding focus groups. Interviews and reviewing documents led to the identification of 10 factors associated with nurses’ available working time. In both educational and non-educational hospitals, the annual working time of all nurses except nurses working in the burn and psychiatric, burn ICU, and pediatric psychiatry wards, which was 1302 h per year, was 1411 h per year. The calculations of the modified WISN method showed that the lowest norm in educational hospitals was for psychiatric, eye surgery, and dermatology wards (0.53) and in non-educational hospitals was for ENT ward (0.57). The highest norm in educational and non-educational hospitals was for burn ICU (3.95) and general ICU (3.07) wards, respectively.

**Conclusion:**

The nursing estimation norm in different wards of the hospital varies, considering that the time available to nurses and their workload in different wards and hospitals are different, and each ward has its special norm therefore, a single norm for all wards and hospitals cannot be used for a fair distribution of nurses.

## Background

The issues of human resource imbalance in healthcare facilities, as well as the justice in the health sector, are important criteria in the distribution of resources and access to healthcare services. Today, they have become one of the main concerns of health system managers in the world, especially in the developing countries [1, 2]. The human resource imbalance in the health sector usually affects the availability, distribution, and performance of human workforce [3]. Given that the healthcare sector is a labor-intensive industry and trained human resources, as the most important data in providing health care, account for the largest share in the health sector expenditure, hence, in most developed countries, the issue of planning human resources has received considerable attention in the health sector [4, 5]. The core of human resource planning is to estimate and predict the number and types of needed human resources to attain the organizational goals, which is the most important human resource management process that is often neglected [6]. Negligence in the human resource planning leads to many problems, including shortages or the excessive human workforce, the unequal human workforce distribution, and inappropriate use of staff in the organization. To prevent the unequal distribution of healthcare providers, some strategies have already been employed, such as the design and implementation of norm estimation of human resource [7, 8].

In the late 1990s, the World Health Organization (WHO) presented the Workload Indicators of Staffing Need (WISN) method to determine the required human resources. The WISN method is a versatile human resource management tool based on the work which is actually undertaken by health sector staff [9]. This method has several advantages including easy to operate, simple to use, technically acceptable, comprehensive, and realistic [10]. Application of WISN to determine the required human resources leads to the establishment of a new norm of the evidence-based human workforce [11]. This method has been used in some countries, such as Bangladesh, Turkey, Uganda, and Indonesia to improve health human workforce planning [3, 9, 12, 13].

Nursing staff planning is of central importance because of the governments’ commitment to patient safety and quality care, as well as the link between nursing workload and patient care quality [14]. In a health care system, among the clinical staff, nurses, who generally account for more than 50% of the total staff in a hospital, play the most important role in promoting community health [15–17]. Therefore, the provision of desirable care services depends on the quantitative and qualitative development of nursing staff. Qualitative development of nursing care is achieved through continuous education, material and spiritual motivation, monitoring, and evaluation of nursing activities. On the other hand, the quantitative development of nursing staff are obtained through the use of appropriate estimation norms of nursing staff and on time supplying and distribution of them in healthcare facilities [18].

In Iran, different norms are used to estimate the number of nursing staff in hospitals. However, a considerable inconsistency in the distribution of nursing staff in different sectors exists due to the lack of a codified standard based on nursing workload indicators. The details of two main proposed models in hospitals are described as follows:

1. Allocation of post approach: In this model, for each hospital bed, 0.85 nursing staff is considered, including matron, nurse, nurse assistant, operation room operator, and anesthesiologist assistant.
2. Systematic approach: In this model, each ward has a special coefficient. To calculate the number of required nurses in each ward, the number of beds is multiplied by the coefficient. For example, the coefficient for internal medicine is 0.74, which means for every two beds 1.5 nurses is needed [8].

Furthermore, in Iran, one of the challenges facing the achievement of health system vision of 2025 is designing the norm estimation of nursing staff in hospitals. It should be mentioned that in Iran, public hospitals are either educational or non-educational. Educational hospitals are those which train health care professionals, such as medical interns [19]. The Ministry of Health and Medical Education of Iran aim to achieve the goals of Iran’s Vision 2025 by putting a compilation of the health care system reform plan on its agenda. Plan means a long-term reformatory program in the health care system including vision, mission, goals, principles, values, policies, and national reformation program [20]. Moreover, the development of national policies for human resources, the crossing from traditional staff management to strategic human resource management, the projection of health sector human resources particularly nursing staff in the hospitals are the other objectives of the Ministry of Health. Therefore, this study was conducted to design the estimation norm of the required number of nurses for educational and non-educational hospitals based on workload indicator in Iran.

### Productivity Improvement Act (PIA)

In Iran to increase efficiency and effectiveness of clinical staffs in healthcare system in both public and private sectors, Productivity Improvement Act (PIA) was passed by the parliament in 2009. Clinical staffs that include nurses, healthcare workers, helping healthcare workers, midwives, technicians and technicians of operating rooms, and anesthesia and medical emergency providing services directly to hospitalized patients benefit from PIA. By implementing this act, the working hour of the target group would decrease from 44 h per week to 36 h based on three factors: the experience of the staff, hard work, and shift work. Night shifts and all shifts on Official holidays are also calculated based on the coefficient of 1.5 [21].

## Methods

### Study design

The present study is a descriptive cross-sectional survey involving focus groups, work study, consensus, interviews, documents and staff records reviews, patient’s record study, and the calculations of modified WISN.

### Study setting

This study was carried out from December 2015 to November 2016 in the Mashhad University of Medical Sciences (MUMS), Iran. MUMS consists of 15 cities and towns, 11 faculties, 29 hospitals (5765 beds), and 14 primary health care (PHC) networks with a total population of 4 960 830.

### Cadre

This study used workload indicator to design an estimation norm to assess the number of nurses required to deliver health services in 49 ward types at two different types of hospitals (educational and non-educational).

### Study procedures

The following methods were used to design the estimation norm:

- Focus group discussions to identify workload components (main, support, and additional activities), the sub-activity for one of the main activities and primary sub-activity standards and allowance standards for support activities and additional activities,
- Work study to test the results of focus group discussions (FGDs),
- A consensus to determine the final sub-activity standards,
- Interview and reviewing documents and staff records to identify factors affecting available working time and calculation of nurses’ available working time,
- Patients’ record study to determine main activity standards, and
- The calculations of modified WISN.

### Study population and sampling

Concerning the fact that according to previous studies, the lack of attention to the nature of the work of each hospitalization ward and the categorization of hospitals (educational and non-educational) was considered as one of the challenges of lack of application of the estimating norms of nursing force in Iranian hospitals [7]. The study population was comprised of 49 ward types in 12 educational and 17 non-educational hospitals affiliated to MUMS, along with the nursing staff working in these hospitals, since the study team expected the designed norms at these hospitals with the highest quality of nursing care could be a model for other similar hospitals, in this study.

Hospitals were selected based on having the best performance in nursing care quality indicators such as the number of hospital infections, mortality rate, and readmission rate.

The members of the focus groups consisted of five to seven experts, knowledgeable and experienced nurses of each ward. The selection criterion was nurses with more than 10 years of nursing experience who worked full time in each ward. Any nurse who met the criteria was considered as a potential participant.

In this study, the nurses with 5 to 15 years’ work experience were selected to measure sub-activities in each ward, given that the workforces were different in terms of capacity, ability, and talent.

In order to determine the standard time of patient care (as one of the main activities) and to estimate the sample size of the required records, 20 patient records were randomly selected from each ward in the 12 months. Preceding the survey and the number of reported sub-activities in the Nursing Report Sheet were identified and multiplied by their standard time. Then, based on the obtained standard deviation from performed nursing care time per in-patient service day, the sample size for each ward was estimated by the following formula:

$$ {n}_o={\left(\frac{z}{d}\cdot \sigma \right)}^2 $$

$$ n=\frac{n_o}{1+{n}_o/N} $$

where *N* is the number of population (all records during the study period), *σ* is population standard deviation (estimated from 20 primary samples), and *d* is the acceptable error rate (5% error is an acceptable error). Hence, *d* is equal to 5% difference in maximum and minimum nursing care time per inpatient service day in the studied wards. Also, *z* is the length of the corresponding point in cumulative probability or the percentile of the standard normal distribution. Accordingly, the sample size was determined, which was 4303 inpatient records (Table 1).

**Table 1:** Sample size (the number of records) needed to determine the final standard time of nursing care from patients by type of ward and hospital

Ward/bed	Sample size (number)		Ward/bed	Sample size (number)	
	Educational hospital	Non-educational hospital		Educational hospital	Non-educational hospital
General surgery	71	57	Cardiology	71	76
Ear, nose, and throat	60	55	Infectious diseases	72	59
Orthopedic	77	70	Pediatric	73	57
Brain and neurosurgery	97	67	Psychiatric	68	NA
Gynecology and obstetrics	77	75	Pediatric psychiatric	63	NA
Urology	62	50	Infant	77	70
Eye surgery	66	NA	Post angiography	82	NA
Corneal transplantation	60	NA	Dermatology	66	NA
Open heart	71	NA	Burn	56	NA
Oral and maxillofacial surgery	74	NA	Gastroenterology	83	NA
Kidney transplantation	77	NA	Lung	56	NA
Liver transplantation	64	NA	Rheumatology	70	NA
Bone marrow transplantation	12	NA	Toxicology	76	NA
Plastic surgery	64	NA	Pediatric kidney	55	NA
CCU	64	79	Pediatric oncology and hematology	64	NA
ICU	78	54	Pediatric surgery	66	NA
NICU	65	50	Pediatric gastroenterology	60	NA
Burn ICU	77	NA	Pediatric neurology	72	NA
PICU	87	NA	Hematology	54	NA
Non-surgery ICU	73	NA	Immunology	50	NA
Surgery ICU	70	NA	Oncology	59	NA
Open heart ICU	72	NA	Cardiovascular surgery	85	NA
Neurology	72	86	Thorax surgery	75	NA
General medical	66	72	Endocrinology	64	NA
Nephrology	53	NA	–	–	–

NA (not available): Indicates the absence of the referred bed in the non-educational hospital

Nurses’ available working time-related factors were identified by interviewing some key informants (*n* = 10 randomly selected matrons) and relevant rules and regulations. The inclusion criterion for recruiting matrons was to have at least 4 years’ management experience, knowledge about factors associated with nurses’ available working time, and the inclination to participate in the study.

### Data collection and analysis

Forty-nine focus groups (*n* = 291 nurses) were held to identify the workload components (main, support, and additional activities), the sub-activity for one of the main activities, sub-activity standards, and allowance standards for support activities and additional activities. One of the main identified activities was patient care (patient care is provided by nurses to patients in a hospital directly and indirectly that recorded in the patient’s record) [22]. Therefore, the group members were asked to identify the sub-activities of patient care, such as injection (subcutaneous, intravenous, intradermal, and intravenous), measure, control, chart vital signs, and serum therapy. Then they were asked to describe all the sub-activities from the beginning to the end of the process, in accordance with the acceptable standards of professionalism. Then nurses participating in the focus groups were asked to set the standard time for each sub-activity (the time it would take for a well-trained and well-motivated member of a particular staff category to perform an activity at acceptable professional standards).

To test the results of FGDs, a work study was used to measure the sub-activity and to ensure the validity and reliability of the FGD results. The work study was repeated three times for each sub-activity. Accordingly, 20 nurses with clinical and research experiences performed the work study using a stopwatch. It should be noted that the selected people for the work study were part of the nursing staff, and the possibility of delaying the activities by the studied nurses in the process of work study was minimized. In order to conclude and finalize the standard time of each sub-activity, the average of the recorded times was compared to the standard times acquired by focus groups per sub-activities. Then, the standard time of each sub-activity was obtained through consensus in the presence of representatives from each focus group. Focus group discussions were analyzed manually by a team of researchers.

To determine the main activity standards, a workshop was conducted by the corresponding author to train nurses of the research team (nurses who were used in the work study) about the process of counting sub-activities in the patients’ records and the importance of the study. In the training session, nurses were provided with several patient records. They were expected to count the sub-activities of these records to assess the nurses’ accuracy in identifying and type of the sub-activities. The errors of nurses were recognized, and they were retrained how to evaluate the patient records. After obtaining the necessary permissions from the hospital, the nurses of the research team referred to the selected hospitals and to the medical records department to study the patient’s records. At this stage, in order to reduce the errors of assessment, two nurses surveyed each patient’s record. If the average patient care standard (nursing care time per day of hospitalization) obtained by each nurse was different, both nurses were asked to resolve the disagreement. The cardiopulmonary resuscitation and transfer out hospital standard time was obtained by examining special forms that record the relevant time from the beginning to the end of the process in the 12 months preceding the survey.

The available work time was calculated by reviewing the documentation of personnel records in the last 24 months. Data of service statistics was collected from the reports and the registers of each hospital management information system.

### WISN variables

The variables of this study included available working time, workload component, activity standard, category allowance standard (CAS), individual allowance standard (IAS), allowance factor, staffing requirement, and staffing estimation norm.

**Fig. 1:**
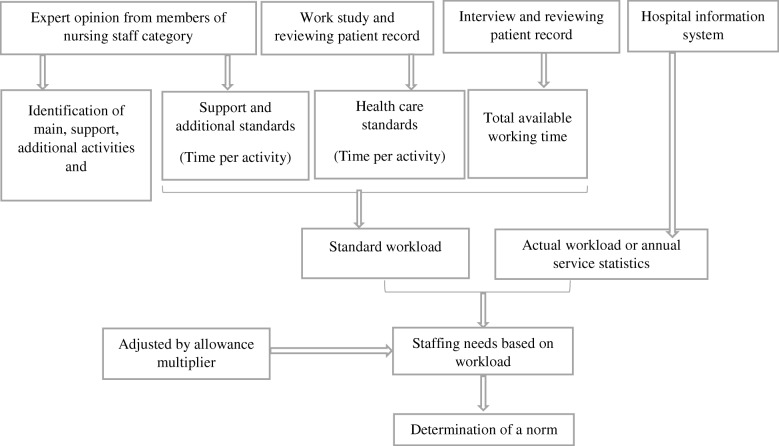
Modified WISN methodology for required nurse estimation

I. Available working time (AWT): A health worker’s time available in 1 year to do his or her job, taking into account authorized and unauthorized absences. In Iran, working days are from Saturday to Thursday. Friday is the official holiday throughout the country. There are three shift schedules for nurses: morning, evening, and night shifts. Morning and evening shifts are 6.5 h daily while night shifts are 12.5 h (excluded from productivity improvement act). In general, the monthly working hours of all employees is 192 h deducted from the non-working days/hours such as annual leave, sick leave, holidays, and administrative leave.
II. Workload component: Workload component is one of the main work activities that takes most of a health worker’s daily working time. There are three types of workload components:
  Health service activity: Health service-related activities performed by all members of the staff category. The annual statistics of health service activity is regularly collected.
  Support activity: Important activities that support health service activities, performed by all members of the staff category. The annual statistics of support activity is not regularly collected.
  Additional activity: Activities performed only by certain (not all) members of the staff category. The annual statistics of additional activity is not regularly collected.
III. Activity standard: The time necessary for a well-trained, skilled, and motivated worker to perform an activity according to professional standards in the local circumstances. There are two kinds of activity standards:
  Service standard: The activity standard for health service activities (Annual statistics are regularly collected for these activities.)
  Allowance standard: The activity standard for support and additional activities. (Annual statistics are not regularly collected for these activities.) There are two kinds of allowance standards:
    Category allowance standard (CAS): Allowance standard for support activities, performed by all members of a staff category.
    Individual allowance standard (IAS): Allowance standard for additional activities, performed by certain (not all) members of a staff category.
IV. Allowance factor: Factor used to take into account the staff requirement of activities. The annual statistics of allowance factor is not regularly collected. There are two kinds of allowance factors:
  Category allowance factor (CAF): Multiplier used to calculate the total number of health workers required for both health service and support activities.
  Individual allowance factor (IAF): Staff requirement to cover additional activities of certain cadre members. IAF is added to staff requirement of health service and support activities.
V. Staffing requirement: The results obtained from the above variables were used to compute the workload-based staffing requirements. In this stage, the annual service statistics from the previous year for every facility/ward are required for calculating the staff requirement.
VI. Staffing estimation norm: Finally, to determine a norm to estimate the required number of nurses in each ward, the required number of nurse staffing from the previous stage was divided by average daily inpatient census (Fig. 1).

It should be noted that according to the objective of the study, the final stage (staffing estimation norm) was added to this work.

### Actions to ensure validity and reliability

The following actions were taken to ensure the quality of the process:

- A researcher team including 20 WISN-trained researchers was employed to collect the data. In addition to WISN training, researchers had sufficient experience in clinical practice and nursing activities. This team was formed to ensure the quality of data collection.
- The original research team monitored each stage of the study closely to ensure the quality of the results. They performed short meetings and reported the summary of the sessions every other day. In this way, we ensured that all the required data were collected.
- Sub-activities related checklists were approved before data entry. Data entry was done on the same day, and any information loss was tracked the next day. The checklist included sub-activity, count, shift, the length of stay, unit number, ward, and hospital.

## Results

Through interviews with matrons and reviewing documents (factors affecting available working time and annual available working time), the results of the FGDs and its test (main, support and additional activities and primary sub-activity standards), the results of the consensus (final sub-activity standards), the results of inpatient’s record study and corresponding calculations (main activity standards) and the output of management information system (service statistics) were identified and determined. Accordingly, the modified WISN and estimation norm was computed.

In general, in Iran, 10 factors influence the available working times of the nurses. These factors were identified and analyzed in this work (Table 2). The average of annual available working days is calculated after incorporating all influential factors. Based on the findings, the available working hours of nurses, except nurses in the burn and psychiatric wards, burn ICU, and pediatric psychiatry, were 1411 h and the working days were 166 days in a year.

**Table 2:** Annual working time calculating for nurses

Row	Factor	Calculations
1	Number of days in a year	365 days
2	Number of Fridays per year	52 days
3	The number of public holidays per year	24 days
4	The average vacation used in a year	18 days
5	The average of sick leave used in a year	3 days
6	The average of maternity leave used in a year	10 days
7	The average of non-employment days for incentive leave, mission, etc. in a year	1 day
8	The average of working days available in a year	365 − (52 + 24 + 18 + 3 + 10 + 1) = 257 days
The average of working hours available annually without decreasing hours due to the Productivity Improvement Act		257 × 7.33 = 1 884 h
9	A deduction of 4 h per week according to Section 2 of the Productivity Improvement Act (4 × 52 = 208)	1 887 – 208 = 1 676 h
	Adjustment of the working hours available under the third section of the Productivity Improvement Act (1.5 times calculated of the nights and official holidays)	0.77 Adjustment factor
10	Considering 40 h of compulsory extra working hours per month (40 × 12 = 480)	1 676 + 480 = 2 156 h
The average of working hours available annually considering the adjustment factor		2 156 × 0.77 = 1 660 h
Hours of nurses’ annual presence at the hospital by applying extra working hours with a deduction of 15% of the working time for allowed unemployment (allowance), rest and personal affairs, fatigue, and delays during work		1 660× 85% = 1 411 h
The average of annual available working days		1 411 ÷ 8.5 = 166 days

### Main activities of nurses and service standards

Patient care, cardiopulmonary resuscitation, and transfer out of the hospital were identified as the main nursing activities. As shown in Table 3, the standard times of caring per patient varied from one ward to another. The service standard for cardiopulmonary resuscitation (CPR) was 90 min, and the service standard for patients transferring from a hospital to other health facilities was estimated as 390 min.

**Table 3:** Service standard of patient care (nursing care per patient day) by type of word and hospital

Ward/bed	Activity standard (min)		Ward/bed	Activity standard (min)	
	Educational hospital	Non-educational hospital		Educational hospital	Non-educational hospital
General surgery	177	165	Cardiology	186	175
Ear, nose, and throat	127	112	Infectious diseases	242	210
Orthopedic	165	135	Pediatric	185	175
Brain and neurosurgery	185	159	Psychiatric	93	NA
Gynecology and obstetrics	167	139	Pediatric psychiatric	135	NA
Urology	172	155	Infant	166	148
Eye surgery	104	NA	Post angiography	183	NA
Corneal transplantation	118	NA	Dermatology	140	NA
Open heart	210	NA	Burn	277	NA
Oral and maxillofacial surgery	148	NA	Gastroenterology	202	NA
Kidney transplantation	256	NA	Lung	197	NA
Liver transplantation	264	NA	Rheumatology	144	NA
Bone marrow transplantation	445	NA	Toxicology	246	NA
Plastic surgery	178	NA	Pediatric kidney	210	NA
CCU	329	295	Pediatric oncology and hematology	219	NA
ICU	424	409	Pediatric surgery	197	NA
NICU	330	325	Pediatric gastroenterology	182	NA
Burn ICU	441	NA	Pediatric neurology	185	NA
PICU	419	NA	Hematology	170	NA
Non-surgery ICU	432	NA	Immunology	185	NA
Surgery ICU	420	NA	Oncology	172	NA
Open heart ICU	445	NA	Cardiovascular surgery	183	NA
Neurology	183	175	Thorax surgery	175	NA
General medical	180	168	Endocrinology	185	NA
Nephrology	179	NA			

### Supportive and additional activities

Table 4 lists the supportive and additional activities. Allowance standards for support and additional activities varied from one ward to another depending on the number of hospitalized patients in the ward. The category allowance factor and individual allowance factor are shown in Table 5.

**Table 4:** List of category allowance factors

Row	Additional activities	Supportive activities
1	Checking the emergency trolley, controlling the ward equipment and alternative for deficiencies	Answering the various questions of patients and his/her family
2	Controlling the medicine shelf and replacing deficiencies	Answering the colleagues’ questions
3	Requesting goods from the warehouse and controlling received items	Teaching the colleagues and students of different disciplines
4	Carrying out interface patient training	Helping colleagues in the ward
5	Carrying out interface training activities for colleague	Answering the phone
6	Performing interface infection control activities	Registering specific information and statistics
7	Performing accreditation interface activities	–
8	Clinical round with supervisor	–
9	Other (division of patients between nurses of the ward, supervision of work of nurses and other workers, registration of leave in attendance and absence system, etc.)	–

**Table 5:** Norm estimation of the required number of nursing staff based on the type of ward and type of hospital

Ward	Type of hospital	Standard workload (patients/year)			CAF*	IAF**	Required nurse (number)			The Norm or coefficient for required nurse estimation
		Patient care	Cardiopulmonary resuscitation	Patient transfer			According to standard workload	By applying category allowance standards	By applying individual allowance standards	
General surgery	Educational	468.85	922.08	212.79	1.18	1.29	38.3	45.19	46.48	0.95
	Non-educational	502.95	922.08	212.79	1.17	0.22	5.9	6.9	7.12	0.88
Ear, nose, and throat	Educational	653.34	922.08	212.79	1.13	0.51	10.91	12.28	12.79	0.67
	Non-educational	740.96	922.08	212.79	1.11	0.02	0.51	0.57	0.59	0.57
Orthopedic	Educational	502.95	922.08	212.79	1.17	1.57	43.11	50.43	52	0.88
	Non-educational	614.72	922.08	212.79	1.13	0.15	3.49	3.96	4.12	0.71
Brain and neurosurgery	Educational	448.58	922.08	212.79	1.19	0.97	30.01	35.85	36.82	1.01
	Non-educational	521.93	922.08	212.79	1.16	0.03	0.8	0.93	0.96	0.86
Gynecology and obstetrics	Educational	496.93	922.08	212.79	1.17	0.97	26.88	31.5	32.47	0.89
	Non-educational	597.03	922.08	212.79	1.14	0.66	15.46	17.62	18.28	0.73
Urology	Educational	482.48	922.08	212.79	1.18	0.88	25.23	29.73	30.61	0.92
	Non-educational	535.4	922.08	212.79	1.16	0.13	3.34	3.87	4	0.82
Eye surgery	Educational	797.95	922.08	212.79	1.1	0.78	13.47	14.83	15.61	0.53
Corneal transplantation	Educational	703.28	922.08	212.79	1.12	0.02	0.55	0.62	0.64	0.6
Open heart	Educational	395.18	922.08	212.79	1.23	0.78	27.05	33.18	33.97	1.16
Oral and maxillofacial surgery	Educational	560.72	922.08	212.79	1.15	0.04	0.9	1.04	1.08	0.8
Kidney transplantation	Educational	324.17	922.08	212.79	1.29	0.23	10.1	13.03	13.27	1.48
Liver transplantation	Educational	214.35	922.08	212.79	1.3	0.06	2.46	3.2	3.26	1.54
Bone marrow transplantation	Educational	186.49	922.08	212.79	1.64	0.33	2.4	3.94	4.27	3.81
Plastic surgery	Educational	466.22	922.08	212.79	1.19	0.15	4.44	5.27	5.42	0.96
CCU	Educational	252.24	922.08	212.79	1.41	0.51	11.78	16.58	17.09	2.12
	Non-educational	281.31	922.08	212.79	1.35	0.55	13.64	18.42	18.79	1.85
ICU	Educational	195.72	922.08	212.79	1.59	0.75	34.68	55.3	56.05	3.05
	Non-educational	202.9	922.08	212.79	1.56	0.47	7.93	12.39	12.86	3.07
NICU	Educational	251.48	922.08	212.79	1.41	0.71	29.09	40.98	41.7	2.09
	Non-educational	255.35	922.08	212.79	1.4	0.37	8.13	11.39	11.76	2.09
Burn ICU	Educational	166.94	818	188.77	1.78	0.14	6.32	11.23	11.38	3.95
PICU	Educational	198.06	922.08	212.79	1.58	0.87	38.5	60.98	61.85	2.98
Non-surgery ICU	Educational	192.1	922.08	212.79	1.61	0.56	22.62	36.49	37.05	3.23
Surgery ICU	Educational	197.59	922.08	212.79	1.59	0.39	13.75	21.8	22.19	3.22
Open heart ICU	Educational	186.49	922.08	212.79	1.64	0.71	31.41	51.61	52.32	3.28
Nephrology	Educational	463.62	922.08	212.79	1.19	0.32	14.75	17.5	17.83	0.97
General medical	Educational	461.04	922.08	212.79	1.19	0.78	24.63	29.26	30.05	1.02
	Non-educational	493.97	922.08	212.79	1.17	0.52	14.52	17.04	17.56	0.9
Neurology	Educational	453.48	922.08	212.79	1.19	0.71	16	19.08	19.78	1.01
	Non-educational	474.21	922.08	212.79	1.18	0.41	9.65	11.41	11.82	0.95
Cardiology	Educational	446.17	922.08	212.79	1.2	0.41	13.32	15.92	16.33	1.06
	Non-educational	474.21	922.08	212.79	1.18	0.47	13.93	16.46	16.93	0.96
Infectious diseases	Educational	342.92	922.08	212.79	1.27	0.48	19.41	22.66	25.14	1.39
	Non-educational	395.18	922.08	212.79	1.23	0.2	6.95	8.53	8.73	1.17
Pediatric	Educational	448.58	922.08	212.79	1.19	0.78	24.2	28.91	29.68	1.01
	Non-educational	474.21	922.08	212.79	1.18	0.54	15.73	18.59	19.13	0.94
Psychiatric	Educational	791.61	818	188.77	1.1	1	22.9	25.23	26.22	0.53
Pediatric psychiatric	Educational	545.33	818	188.77	1.15	0.12	3.98	4.59	4.71	0.79
Infant	Educational	499.92	922.08	212.79	1.17	0.27	3.77	4.41	4.69	0.91
	Non-educational	560.72	922.08	212.79	1.15	0.32	4.87	5.6	5.92	0.81
Post angiography	Educational	453.48	922.08	212.79	1.19	0.4	9.04	10.78	11.18	1
Dermatology	Educational	838.25	922.08	212.79	1.15	0.36	4.13	4.52	4.88	0.53
Burn	Educational	265.78	818	188.77	1.38	0.4	18.02	24.85	25.25	1.93
Gastroenterology	Educational	410.83	922.08	212.79	1.22	0.36	18.07	21.98	22.24	1.1
Lung	Educational	421.25	922.08	212.79	1.21	0.32	15.43	18.67	18.98	1.07
Rheumatology	Educational	576.3	922.08	212.79	1.15	0.32	11.6	13.28	13.6	0.75
Toxicology	Educational	337.35	922.08	212.79	1.28	0.79	32.61	41.62	42.41	1.42
Pediatric kidney	Educational	395.18	922.08	212.79	1.23	0.44	12.06	14.79	15.23	1.17
Pediatric oncology and hematology	Educational	378.94	922.08	212.79	1.24	0.96	34.76	43.06	44.01	1.22
Pediatric surgery	Educational	421.25	922.08	212.79	1.21	1	32.45	39.25	40.25	1.08
Pediatric gastroenterology	Educational	455.97	922.08	212.79	1.19	0.26	7.68	9.14	9.39	0.99
Pediatric neurology	Educational	448.58	922.08	212.79	1.19	0.27	8.47	10.12	10.39	1.01
Hematology	Educational	488.16	922.08	212.79	1.18	0.35	14.69	17.27	17.62	0.91
Immunology	Educational	525.24	922.08	212.79	1.16	0.06	1.64	1.9	1.96	0.83
Oncology	Educational	482.48	922.08	212.79	1.18	0.29	12.87	15.16	15.46	0.91
Cardiovascular surgery	Educational	453.48	922.08	212.79	1.19	0.66	32.81	39.1	39.76	0.98
Thorax surgery	Educational	474.21	922.08	212.79	1.18	0.27	12.03	14.22	14.49	0.96
Endocrinology	Educational	448.58	922.08	212.79	1.19	0.15	6.9	8.24	8.39	0.99

*CAF is a multiplier that is used to calculate the total number of health workers, required for both health service and support activities

**IAF shows required nurse number to perform additional activities

### Estimation norm of nurses required in wards of the hospital

Table 5 illustrates the standard workload, category allowance factor, and individual allowance factor required nurse calculations for each ward of educational and non-educational hospitals. The coefficient in the final column shows the estimation norm of the required nursing staff based on the type of inpatient ward and type of hospital. For example, the norm for general medical ward in non-educational hospital is 0.9, which means for every 10 beds/patients, nine nurses are needed in a year. As Table 5 shows, in educational hospitals, the most estimation-nursing norm is related to burn ICU (3.95), bone marrow transplantation (3.81), and open-heart ICU (3.28). On the other hand, in non-educational hospitals, it is related to general ICU (3.07), NICU (2.09), and CCU (1.85). In educational hospitals, the lowest coefficient belongs to the eye surgery section, psychiatry unit, dermatology (0.53), eye transplantation unit (0.6), and ENT (0.67). In non-educational hospitals, this category is related to ENT (0.57), orthopedic (0.71), and obstetrics and gynecology unit (0.73).

## Discussion

Given the increasing demand for health care services on the one hand and the relationship between the quality of services and the number and mix of the professional workforce needed by health centers on the other, in this study, hospitals with the best performance in providing standard care were considered to estimate the required nursing force. Similarly, Namganda et al. chose hospitals with the best-quality services to determine the level of staff required for hospitals affiliated with Uganda Catholic Medicine Bureau [23]. Regardless of the method, reference standards are needed to calculate workforce requirement. Situation observed in a facility or region or part of the country can be set as a standard [24].

In this study, patient care, patient transfer out hospital, and cardiopulmonary resuscitation were identified as the workload components of the nurses’ main activities. In the quality-activity-dependence-based nursing staff planning, it is assumed that patient dependency is a precise scale of patient’s need for nursing care, and the need for human resources based on patient dependency has a relatively high accuracy [25]. In Western Australia, nursing hours per patient day (NHPPD) are used as a priority in determining the need for nursing staff [25]. In this study, the transferring patients are another part of the nursing workload. Patient transfer is inevitable and is a part of the treatment process [26], which is intended to improve the provision of health services and reduce mortality and disability in patients [27]. In Iran, given the patient’s condition, the trained nurse is designated as a member of the patient’s transfer team in consultation with the referring and receiving physician [28]. Hence, given that a relatively large number of patients are transferred to other health facilities to receive higher-level services, this has led to a great pressure for the health system including excessive use of human resources, especially nurses.

In the present study, CPR is another component of the workload for the main activities of nurses. Today, all hospitals have a CPR team that employs advanced technology during cardiopulmonary arrest [26]. Although patient resuscitation is carried out by different members of the rescue team with different specialties, the important role of nurses in the team of recovery and identification of patients with critical conditions and prevention of cardiac arrest is inevitable. Also, they have a crucial task in performing a timely and principled resuscitation and post-care support to improve the side effects of the recovery in the hospitalized patients. Since nurses are among the first members of the rescue team, they play a very critical role in the life of the patient [27].

In this study, the available working time for nurses in four burn wards, burn ICU, pediatric psychiatry, and psychiatry was less than that in other wards due to the productivity improvement act. Hence, staff working in those wards benefit from one extra month’s paid leave which cannot be stored and must be used during the year [21].

The result showed that the nurses’ working days were 166 days yearly. Hossain and Alam calculated that nurses work for 205 days per year in Bangladesh [12], while nurses work for 199 days in Turkey as reported by Ozcan and Hornby [9]. In this regard, Shipp used 228 days from the 248-day domain [10]. In 2004, Namagandar used 246 days in Uganda [23], and Mugisha obtained 227 days in Uganda in 2008 [13]. The findings of the current study and similar studies indicate that the available working time depends on the working conditions of the country and the organization under study.

In addition, in this study, we deigned the estimation norm for the number of required nursing staff in hospitals wards. The results showed that considering the various average time of nursing care per patient day in different wards and according to the type of the hospital (educational or non-educational), the coefficients of estimation of nursing staff is different than other studies [18, 29, 30]. Based on the system-oriented approach, the coefficient for all ICUs and neonatal ICUs is 4.04 and 2.9, respectively [18]. In another study, the estimation norm of nursing staff of 3.13 was obtained for all ICUs [30]. Bahadori et al. used a coefficient of 3.47 for all ICUs in their study [29]. However, the results of this study showed that due to the amount of nursing care needed in the intensive care units, a single coefficient could not be considered. According to the results, each of the specialized wards in the pediatrics hospital had its own nursing estimation coefficients; while based on the systematic approach, the selected coefficient for all specialized wards in the children’s hospital were equal to 0.9 [18]. In similar studies, the coefficient to estimate the required number of nurses needed in the pediatric surgery ward was 0.97 and 0.88 [29, 30]. In the present study, for the ophthalmology, nephrology, endocrinology, and bone marrow transplant sections, coefficients of 0.6, 1.48, 1.54, and 3.81, were obtained, respectively; while based on the system-oriented approach, the coefficient of 2.9 is proposed for all transplant wards [18]. Moreover, based on the systematic approach, the proposed coefficient for CCU, burn, and neurosurgery was 2.9, while these wards did not have the same coefficients in the present study. Several reasons contribute to this difference. In contrast to the previous studies, we used an evidence-based method based on the workload indicator to calculate the required number of nursing staffs. Another reason could be the new rules made by Iran’s health system, which resulted in the reduction of the working hours of nurses. In addition to that, the increase in the maternity leave from 6 to 9 months was taken into account in the calculation of nurses’ available time.

When the health care centers run into a severe shortage of professional staff due to lack of financial resources, strategies such as task shifting and the use of nursing students are raised to compensate this shortage. Using these strategies raises concern about the quality of nursing care [3]. In Iran, task shifting of the nursing staffs has been carried out with the training and employing of nurse assistant (called Behyar in Farsi) over the past years [31]. In 2012, the use of nursing students in hospitals was suggested by the Ministry of Health [32]. It is obvious that the implementation of such plans leads to a reduction of pressure on the nurses. The reuse of the WISN method is useful in the review of the nursing estimation norms.

### Limitations

Since the WISN method uses the annual service statistics to measure workload and the accuracy of the WISN method and results are determined by the accuracy of the statistics, if the statistics have not been recorded properly, then WISN results will be in error. Moreover, the WISN method uses the statistics of healthcare services of the last year; therefore, this method calculates the number of the workforces that was needed for last year. This is not usually a serious and disturbing issue due to the slow changes in the workload of health care centers in line with demographic changes and economic conditions. To solve this problem, the latest and the most current workload information was used. Occasionally, the lack of medication or supplies reduces the workload of a facility. If the shortfalls are not considerable, their effects can be ignored. In the present study, it was tried to reduce the effect of this problem by selecting educational and non-educational hospitals with desirable nursing care. Another limitation of this study was that our observations might have been affected by the human error or violations in measuring the time. Since selected nurses were responsible for this type of measuring in the work study, this effect was negligible. In this work, only hospitals affiliated to MUMS were studied which makes it difficult to generalize the results to other hospital throughout the country. Considering that the average time of nursing care rarely varies in the same sectors and since the hospitals with the highest level of care were considered in the present research, this issue was not a concern. The last limitation of the study was the fact that we relied on experts’ views to estimate some activity standards; this might have affected the precision of the estimation.

## Conclusion

According to the healthcare system reform plan, the health sector of Iran is constantly under pressure to increase efficiency and equality. Proper human resource management and planning can contribute greatly to improve the efficiency and equity in the health care sector. WISN has been estimated for nurses working in different inpatient wards of educational and non-educational hospitals in Iran. The different coefficient of WISN indicates that a general and constant coefficient cannot be used to estimate the required number of nursing staff.

The result of a study can be used as a tool for auditing the distribution of nurses throughout the country. The use of required nurse estimation norms based on the workload indicators along with eliminating the intuitive decisions of managers can be considered as an effective strategy to improve both nursing staff and patient care levels. Although the application of these norms initially increases staff costs due to the increased nursing staff, the overall costs are reduced by improving both the nurses and patients satisfaction and reducing the number of nurses resignation and eliminating its harmful consequences. Briefly, planning for nursing personnel can be done using WISN method for proper allocation and deployment; therefore, the workload can be distributed among nursing staff in all hospitals to improve the health care services.

## Abbreviations

CCU: Critical care unit
ENT: Ears, nose, and throat
FGD: Focus group discussion
ICU: Intensive care unit
MUMS: Mashhad University of Medical Sciences
NHPPD: Nursing hours per patient day
NICU: Neonatal intensive care unit
PHC: Primary health care
PIA: Productivity Improvement Act
WHO: World Health Organization
WISN: Workload Indicators of Staffing Needs
